# Morphological and genetic evaluation of the thumbprint emperor, *Lethrinus harak* (Forsskål, 1775) in the Pacific and Indian Oceans

**DOI:** 10.12688/f1000research.23740.2

**Published:** 2021-03-16

**Authors:** Muhammad Afrisal, Yukio Iwatsuki, Andi Iqbal Burhanuddin

**Affiliations:** 1 Department of Fisheries Science, Faculty of Marine Science and Fisheries, Hasanuddin University, Makassar, Indonesia; 2 Division of Fisheries Sciences, Faculty of Agriculture, Miyazaki University, Miyazaki Prefucture, Japan; 3 Marine Biology Laboratory, Faculty of Marine Science and Fisheries, Hasanuddin University, Makassar, Indonesia

**Keywords:** Lethrinus harak, morphometric characters, principal component analysis, genetic distance, Cytochrome Oxidase Subunit I

## Abstract

**Background**: The Lethrinidae (emperors) include many important food fish species. Accurate determination of species and stocks is important for fisheries management. The taxonomy of the genus *Lethrinus* is problematic, for example with regards to the identification of the thumbprint emperor *Lethrinus harak. *Little research has been done on *L. harak* diversity in the Pacific and Indian Oceans. This study aimed to evaluate the morphometric and genetic characters of the thumbprint emperor, *L. harak* (Forsskål, 1775) in the Pacific and Indian Oceans.

**Methods**: This research was conducted in the Marine Biology Laboratory, Faculty of Marine Science and Fisheries, Hasanuddin University, and Division of Fisheries Science, University of Miyazaki. Morphometric character measurements were based on holotype character data, while genetic analysis was performed on cytochrome oxidase subunit I (COI) sequence data. Morphometric data were analysed using principal component analysis (PCA) statistical tests in MINITAB, and genetic data were analysed in MEGA 6.

**Results**: Statistical test results based on morphometric characters revealed groupings largely representative of the Indian and Pacific Oceans. The Seychelles was separated from other Indian Ocean sites and Australian populations were closer to the Pacific than the Indian Ocean group. The genetic distance between the groups was in the low category (0.000 - 0.042). The phylogenetic topology reconstruction accorded well with the morphometric character analysis, with two main *L. harak* clades representing Indian and Pacific Ocean, and Australia in the Pacific Ocean clade.

**Conclusions**: These results indicate that the morphological character size of *L. harak* from Makassar and the holotype from Saudi Arabia have changed. Genetic distance and phylogeny reconstruction are closely related to low genetic distance.

## Introduction

The Lethrinidae, and in particular the genus Lethrinus, comprise many fish species of importance in commercial and subsistence fisheries around the worldp ^1^. One important fisheries target species, the thumbprint emperor, *Lethrinus harak* (Forsskål, 1775), is widely distributed throughout the Indo-West Pacific from the Red Sea and East Africa to Samoa, and from north Japan to northeastern Australia ^2^. This fish tends to be found living solitary or in small schools over shallow sandy areas and coral rubble, among mangroves, in lagoons and channels, and in seagrass areas inshore and adjacent to coral reefs, at depths ranging from 1m to 20m ^3, 4^.

Sustainable fisheries management should be based on an understanding of fish biology and ecology, including the accurate determination of species and stocks ^5^. Research on the life history, biology and genetics of *L. harak* has been conducted on populations in both the Indian and Pacific Oceans. Like the majority of Lethrinid species. *L. harak* is a relatively long-lived fish; however, although studies on several Pacific Ocean populations have provided information on the reproductive biology of *L. harak* in Japan, the Philippines and Indonesia ^6^, many aspects of the reproductive biology of this species are still unknown ^7^. Genetic studies on *L. harak* have reported the possible presence of cryptic species in the southwest Indian Ocean (SWIO) ^8^. Genetic information indicates the possible presence of two clusters, in the Indian Ocean and the Pacific Ocean. However, to date there are no studies evaluating morphological and genetic characters of *L. harak* across the Indian and Pacific Oceans.

Combined analyses of morphometric characters and genetic variation are increasingly common. Such methods aim to evaluate the differences and similarities in the grouping of individuals and populations using measures such as similarity indices, genetic distances and cluster analysis ^9^. Species identification based on morphometric and meristic characters should be supported by molecular identification methods, as identification based solely on morphometric-meristic characters can often lead to misidentification, taxonomic ambiguity, and fluctuations in the number of recognised species. Such studies have found strong indications of separated populations with sufficient differences to warrant the designation of new species ^10^ or, conversely, to indicate that currently recognised species are in fact synonyms ^11^. These characters can be related to food habits and homoplasy (different species develop, rather than inherit, similar traits), as some traits can be lost or emerge depending on the environment. Such phenomena are enabled by phenotypic and genotypic plasticity and can lead to the presence of cryptic diversity) and hidden species, as well as considerable variations in colour patterns and other features between different life-stages of the same species ^12^.

Information on the population structure of *L. harak* based on morphometric and genetic characters is needed to evaluate the stocks of this fish. The genetic structure of the population can be studied based on mitochondrial DNA (mtDNA). Although mitochondria undergo rapid evolution, the cytochrome oxidase subunit I (COI) is a segment of mtDNA with low evolution (conserved region) and has been chosen as a widely used genetic marker in so-called COI barcoding ^13^. This study aimed to evaluate the relationship between *L. harak* in the Pacific Ocean and the Indian Ocean based on morphological characters and genetic diversity using a standard COI mtDNA genetic marker.

## Methods

### Ethical statement

This research was conducted for three months (November 25, 2019 – February 24, 2020) under the Enhancing International Publication Program conducted by the Directorate for Human Resource Qualifications Directorate General of Resources for Science, Technology & Higher Education Ministry of Research, Technology and Higher Education of the Republic of Indonesia in collaboration with the University of Miyazaki. This study complied with relevant ethical regulations in Japan and Indonesia. In Indonesia, the doctoral research proposal of the first author was assessed and accepted by an academic panel. This assessment included compliance with institutional ethical guidelines in vigour at Universitas Hasanuddin. A letter of acceptance was issued by the Head of the Laboratory of Fisheries Sciences, Faculty of Agriculture, Miyazaki University, permitting the research activities at the University of Miyazaki's Division of Fisheries Science Museum (MUFS). The issuance of this acceptance was based on the research proposal, including compliance with institutional ethical guidelines in vigour at Miyazaki University. The use of the samples in this study did not require specific ethical approval for the following reasons: all fish specimens used were already dead when they were acquired; there was no use of live animal specimens; specimens obtained from fish port auction houses had been captured by legal fisheries and were obtained following all applicable regulations; the IUCN Red List assessment lists *Lethrinus harak* in the Least Concern category (not considered at risk of extinction); this study was based on field research (surveys) with no experimental component.

### Fish specimens

Specimens of *L. harak* were selected as paratypes at the Marine Biology Laboratory, Faculty of Marine Science and Fisheries, Universitas Hasanuddin (MSFUH), and the Division of Fisheries Science, Miyazaki University. A total of three specimens for morphometric measurements namely MSFUH000591, MSFUH0000592, MSFUH0000593, and only one specimen is taken for DNA barcoding (MSFUH000591), collected from the Makassar Strait (-4°58’N, 119°17’17’’E), were obtained from the Paotere fish auction site, Makassar City, South Sulawesi, Indonesia. The samples were placed in a coolbox with ice for transport to the Marine Biology Laboratory, Universitas Hasanuddin, Makassar. The samples were cleaned under running water to remove any dirt and organic matter attached the exterior of the specimens. Each sample was placed on its right-hand side and sterilised using alcohol. A fin-clipping of around 1.8cm in length was taken from the pectoral fin and inserted into a 2 ml micro tube 2 filled with 90% alcohol. Each sample was numbered and labelled according to the Marine Science and Fisheries Universitas Hasanuddin (MSFUH) catalogue number system, preserved in 10% formalin and stored in the laboratory collection. 

**Figure 1.**
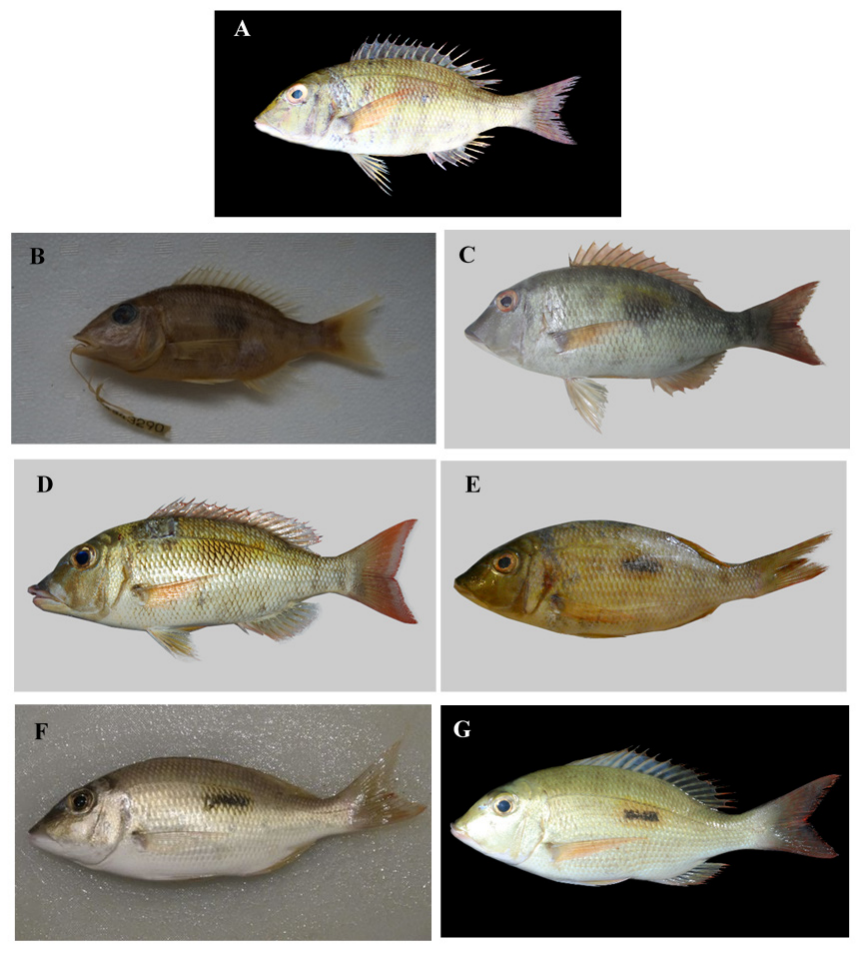
Specimens of *Lethrinus harak*. **A.** Specimen JN311937, paratype, SL 170mm, collected from Arumbai Fish Market (-3°68’N, 128°18’E), Ambon, Indonesia, 06 January 2016 (photographed by Limmon, reproduced from BOLD under a CC BY-NC-SA 3.0 license). **B.** Specimen MUFS43290, paratype, SL 131mm, from the MUFS (Miyazaki University, Fisheries Sciences) museum collection (photographed by the authors). **C.** Specimen MSFUH000591, paratype, SL 217mm, collected in the Makassar Strait (-4°58’N, 119°17’17’’E), 20 January 2019 (photographed by the authors – Muhammad Afrisal). **D.** Specimen HM423533, paratype, SL 219mm, collected from Lizard Island (-14°66’N, 145°44’E), Queensland, Australia, 07 September 2008 (photographed by the Australian Museum team, Sydney, modified from BOLD under a CC BY-NC-SA 3.0 license). **E.** Specimen HQ561476, paratype, collected in Mozambique (-22°84’N, 35°55’E), 15 November 2010 (photographed by Connell, modified from BOLD under a CC BY-NC-SA 3.0 license). **F.**Specimen MH331781, holotype, SL 200mm, collected from Thuwal, Mecca, Saudi Arabia (22°30’N, 39°09’N), 22 March 2017 (photographed by catta, modified from GBIF under a CC BY-NC 4.0 license). **G.**Specimen JQ350086, paratype, SL 190mm, collected from Nosy Tanikely-West, Quest (-13°48E, 48°24N), Antananarivo, Madagascar, 07 May 2008, (photographed by Planes et al., 2008, modified from BOLD (no rights reserved)).

The seven specimens measured in Japan formed part of the pre-existing fish collection of the University of Miyazaki Division of Fisheries Science Museum (MUFS). These specimens had been collected from Yonashiro, Uruma, and Okinawa, Japan, with catalogue and sample numbers based on the cataloguing system of Miyazaki University Department of Fisheries Science (MUFS): MUFS 43290, MUFS 4417, MUFS 6025, MUFS 8819, MUFS 12632, MUFS 2098, and MUFS 6014. One specimen had been collected from Meitsu, Nago, Miyazaki, Japan (MUFS 16284) and one from the Philippines (MUFS 6136) ( Figure 1).

The preceding samples were used in this study to represent the Pacific Ocean. The samples used to represent the Indian Ocean were downloaded from the Global Biodiversity Information Facility (GBIF) and The Barcode of Life Data System (BOLD): KS66045, HQ561476, MH331781, dan JN311937. Measurements of these remotely sourced specimens were made using ImageJ version 1.52a software ^14^, except for the interorbital width character, which could not be measured from the downloaded photographs. The morphological characters were compared with reference to data from three holotypes: MNHN 9087, holotype of *L. azureus*; RMNH 5758, holotype of *L. rhodopterus*; and BMNH 1873.3.160, holotype of *L. bonhamensis* ^15^. Measurement of morphometric characters using conventional methods by following the holotype character *L. harak* included standard length, body depth, head length, pectoral length, pelvic length, orbital length, interorbital width, snout length, suborbital width, upper jaw length.

### DNA extraction

DNA extraction specimen Makassar was carried out using DNeasy Blood and Tissue Kit (Qiagen, Cat. No. 69504) following the manufacturer’s protocols. Fin clippings (1.8cm) were taken from the pectoral fin of each specimen using surgical scissors or a surgical scalpel. Each sample was preserved in a labelled 2ml tube filled with 95% alcohol.

A sub-sample weighing 0.025mg was taken from each fin clipping sample and placed in a 2mL tube to which 180µL of ATL solution (tissue lysis buffer) and 20µL of proteinase K were added. The tubes were vortexed and incubated at 56°C for 1–3 hours or overnight. Then 200µL AL buffer (lysis buffer) solution and 200µL ethanol were added before vortexing and centrifuging at 8000rpm for 1 minute. The supernatant was transferred to a new tube, 500 µL AW1 (column wash buffer 1) was added, and the tube was centrifuged for 1 minute at 8000rpm. This step was repeated with AW2 (column wash buffer 2) and centrifuged at 14,000rpm for 1 minute. The column was transferred to a new 1.5mL tube and 100μL warm AE buffer was added, then centrifuged for 1 minute, incubated for 10–15 minutes, and centrifuged at 6000×g (8000rpm) for 1 minute. RNAse was added to the extracted DNA solution and incubated for 24 hours. The extracted DNA was prepared for use through dilution, at a ratio of 30µL DNA and 70µL ddH _2_O.

### Electrophoresis

Agarose gel was prepared by mixing 1.4g of agarose 0.8% in 180mL of 1x TAE buffer solution. A colouring agent (1.5μl GelRed) was added to the gel while still warm and liquid before pouring it into the mould. Aliquots of 2μL DNA template were pipetted and mixed with 1μl of DNA loading dye solution and loaded into the wells in the agarose gel. Electrophoresis was performed at 100 volts for 90 minutes. The results were visualised using an ultraviolet transilluminator (biostep Dunkelhaube, bi000052).

### DNA amplification

The DNA barcode analysis used a segment of mtDNA from the cytochrome b oxidase subunit 1 (COI) gene. The target sequences were amplified using the primer pair developed by Ward *et al.* ^16^: Fish F1-5’ TCA ACC AAC CAC AAA GCA TTG GCAC 3 (forward) and Fish R1-5’ TAG ACT TCT GGG TGG CCA AGA ATCA 3 (reverse). Each reaction tube contained 5µL of PCR mix Hotstart (Cat. No. 203645, QIAGEN GmbH, QIAGEN Strasse 1, 40724 Hilden, Germany), 3µL DNA template, 3µL ddH2O, and 0.675µL each of the forward and reverse primers. The PCR amplification procedure (Labcycler Gradient, 012-102, Sensoquest) comprised the following stages: initial denaturation at 95°C for 5 minutes; 35 cycles with denaturation at 94°C for 1 minute, annealing at a primer-specific temperature for 1 minute, elongation at 72°C for 1 minute; and final elongation at 72°C for 10 minutes. The amplified PCR products were stored in a freezer (-20°C) until the separation process. The annealing temperature followed a gradient of ±5°C compared to the reference temperature for the primer pair, in order to determine the optimal annealing temperature, resulting in a bright and sharp band under electrophoresis. PCR products that have been tested positive for a length of 682 bp, then proceed with the sequencing of nucleotides to obtain DNA sequences. The sequencing was carried out at the company 1st Base Asia, Singapore using BigDye® Terminator ver.3.1 cycle sequencing KIT and an automated HiSeq-X sequencing machine. The primers used for the sequencing process are the same as the primers used for the PCR amplification process.

### Data analysis

Principal component analysis (PCA) was used to analyse the morphometric data, and was implemented in MINITAB version 17 ^17^. The quality of the DNA sequences obtained was evaluated using the software Sequence Scanner 2.0 (Applied Biosystems, USA). The sequence data obtained from this study were complemented by additional sequences downloaded from the National Center for Biotechnology Information (NCBI) and DNA Data Bank of Japan (DDJB) databases (accession numbers: HQ561476 Mozambique; KF489627 South Africa; JQ350086 Madagascar; MH331781 Saudi Arabia; JF952781 Japan; JN311937 Indonesia; HM423533 Australia). The forward and reverse sequence data were combined using the software Bioedit version 7.2.6.1 ^18^. The sequences were trimmed and aligned using the ClustalX ^19^ routine in MEGA 6 ^20^. The phylogenetic topology reconstruction was implemented in MEGA 6 with the neighbour-joining method and genetic distance was analysed using the compute within groups mean distance option.

Although PCA in this research was implemented in MINITAB, the free software JASP ^21^ could be used as an alternative, although we could not guarantee total equivalence between the two software packages. Genetic analyses in this study were implemented using Sequence Scanner 2.0, ClustalX 2.1 and MEGA 6.

## Results

### Morphological characters

**Figure 2.**
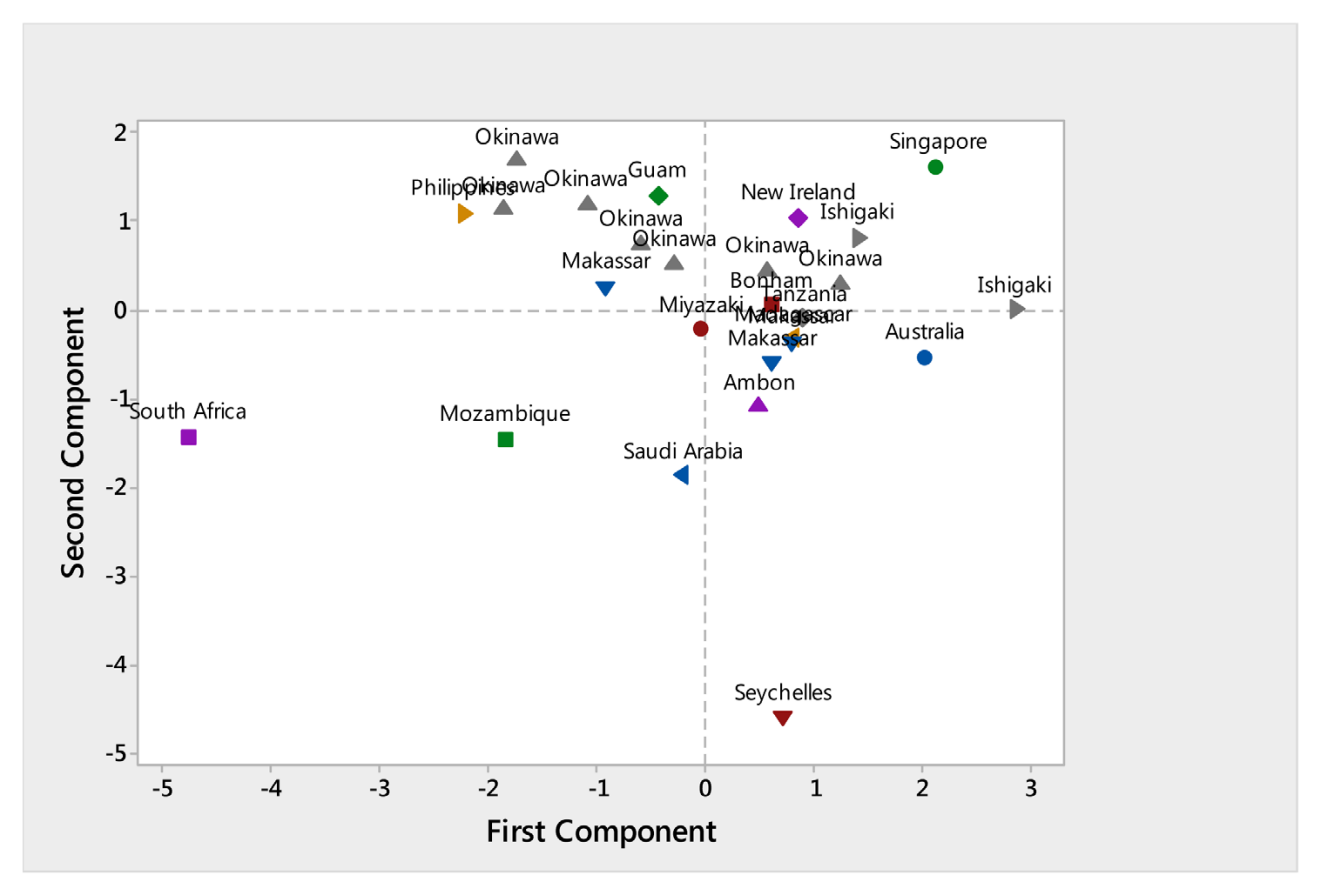
Distribution of morphological characters of *Lethrinus harak* populations with respect to the two principal components (axes) of the principle component analysis.

The PCA analysis shows that the distribution of morphological characters of individual *L. harak* specimens (see Table 1) indicates a general pattern of close relationships within two population groups: those in the Pacific Ocean and those in the Indian Ocean ( Figure 2). Holotype specimens used in this research (Bonham, New Ireland, Singapore) tend to be grouped to the right above the X axis ( Figure 2).

**Table 1.** Morphological characters of *L. harak* holotypes and paratypes in the Pacific and Indian Oceans.

Catalogue number	Locality	Standard length (mm)	Inverse of ratio to standard length				Inverse of ratio to head length				
			Body depth	Head length	Pectoral length	Pelvic length	Orbit length	Interorbital width	Snout length	Suborbital width	Upper jaw length
**Holotype**											
MNHN 9087	New Ireland	240	3.0	3.0	3.6	4.1	3.8	3.6	2.0	3.0	2.4
RMNH 5758	Singapore	283	2.9	2.9	3.4	5.4	4.2	4.0	1.9	2.8	2.6
BMNH 1873.3.160	Bonham	207	2.8	3.1	-	4.0	3.9	3.6	1.9	2.9	2.7
**Paratype**											
UG 1783	Guam	170	2.9	3.0	3.1	4.4	3.7	3.5	2.0	3.1	2.7
UMUTZ 52628	Ishigaki	225	2.9	3.4	3.4	4.6	4.1	3.4	2.0	3.1	2.6
UMUTZ 52510	Ishigaki	285	2.9	3.1	3.3	4.2	4.2	3.5	1.8	2.7	2.5
MUFS 6136	Philippines	120	2.8	2.9	3.5	4.4	3.4	3.4	2.2	2.9	2.7
MUFS 43290	Okinawa	131	2.6	2.8	3.3	4.7	3.6	3.6	2.0	2.9	2.8
MUFS 4417	Okinawa	143	2.8	3.1	3.6	4.6	3.5	3.8	2.1	3.3	2.7
MUFS 6025	Okinawa	132	2.9	3.1	3.5	4.3	3.5	3.5	2.1	3.0	2.6
MUFS 16284	Miyazaki	189	2.7	3.2	3.3	4.3	3.7	3.1	2.0	2.7	2.7
MUFS 8819	Okinawa	176	2.6	3.1	3.8	4.4	4.4	3.6	1.9	2.9	2.7
MUFS 12632	Okinawa	177	2.9	3.0	3.3	4.4	3.3	3.5	1.9	3.0	2.9
MUFS 2098	Okinawa	247	2.8	2.9	3.0	4.0	3.9	3.4	1.8	2.8	2.5
MUFS 6014	Okinawa	240	2.7	3.0	3.4	4.4	3.3	3.2	1.9	2.9	2.5
MSFUH591	Makassar	217	2.7	3.0	3.5	4.1	3.9	3.4	1.9	2.6	2.8
MSFUH912	Makassar	209	2.8	3.1	3.4	4.6	3.9	3.8	2.0	2.5	2.9
MSFUH593	Makassar	163	2.8	2.9	3.1	4.0	3.5	4.5	2.1	2.6	2.5
KS66045	Seychelles	265	2.8	3.2	3.0	4.0	3.7	-	1.9	2.3	4.0
HQ561476	Mozambique	185	2.7	3.3	3.4	5.4	3.4	-	2.3	2.8	2.8
MH331781	Saudi Arabia	200	2.7	3.3	3.2	4.5	3.8	-	2.0	2.8	3.0
JN311937	Ambon	170	2.9	3.3	3.2	4.4	3.8	-	2.0	2.7	2.7
HM423533	Australia	219	3.0	3.3	3.4	4.9	3.8	-	1.9	2.6	2.3
FC86965	Tanzania	200	2.6	3.8	3.1	4.7	3.2	-	2.1	2.3	2.5
JQ350086	Madagascar	235	2.8	3.3	3.3	4.2	3.5	-	2.0	2.6	2.4
KF489627	South Africa	215	2.7	3.2	3.6	5.4	3.3	-	2.4	3.4	3.0

In general, individuals from Pacific Ocean populations, i.e. from Japan (Okinawa, Miyazaki, and Ishigaki), Indonesia (Makassar and Ambon), the Philippines, and Australia, are predominantly grouped in the upper quadrants of the PCA graph ( Figure 2).

Populations from the Indian Ocean were mostly spread across the lower left quadrant, i.e. South Africa, Mozambique, and Saudi Arabia, with the Seychelles being the only population placed (very low) in the bottom right quadrant. However, the specimens from Tanzania and Madagascar grouped together with the Pacific Ocean populations in the upper right quadrant and had morphometric characters similar to the holotype of *L. harak.*

### Genetic distance and phylogenetic topology reconstruction for L. harak

The genetic distance analysis using the compute within groups mean distance method indicates that, in general, *L. harak* populations in the Pacific Ocean and the Indian Ocean have a close kinship relationship ( Table 2). The molecular analysis in this study shows that the smallest genetic distance between populations in the Indian Ocean (South Africa and Mozambique) is 0.000, while the lowest genetic distance in the Pacific Ocean (between Japan and Indonesia) is 0.004. The lowest genetic distance between populations in the two oceans (Saudi Arabia and Indonesia) is 0.031. Meanwhile the largest genetic distance is 0.042 (between Madagascar and Japan, Makassar, and Australia), and is still included in the low category.

**Table 2.** Genetic distance between *L. harak* populations based on the compute within groups mean distance method.

No	Catalogue number	Locality	1	2	3	4	5	6	7	8
1	HQ561476	Mozambique	-							
2	KF489627	South Africa	0.000	-						
3	JQ350086	Madagascar	0.004	0.004	-					
4	MH331781	Saudi Arabia	0.004	0.004	0.007	-				
5	JF952781	Japan	0.038	0.038	0.042	0.034	-			
6	MSFUH0000591	Makassar	0.038	0.038	0.042	0.034	0	-		
7	JN311937	Ambon	0.034	0.034	0.038	0.031	0.004	0.004	-	
8	HM423533	Australia	0.038	0.038	0.042	0.036	0.009	0.009	0.005	-

**Figure 3.**
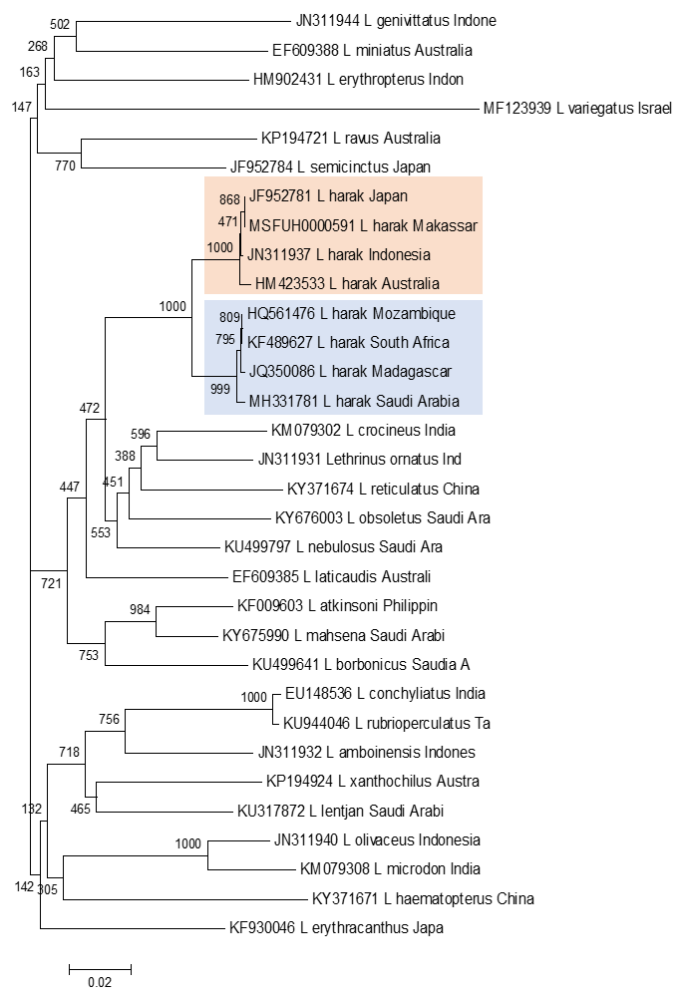
Phylogenetic reconstruction for eight Pacific and Indian Ocean *L. harak* populations, nested within the genus *Lethrinus*, using the neighbour-joining method with the Kimura 2-parameter model and bootstrapping with 1000 replicates.

Phylogenetic topology reconstruction was made to confirm the morphological grouping and genetic distance between the Pacific and Indian Oceans. The phylogenetic reconstruction ( Figure 3) used the neighbour-joining method with outgroup sequence data obtained from GenBank for species in the genus *Lethrinus* found in eight populations in the Indian and Pacific Oceans.

The topology of this neighbour-joining phylogenetic reconstruction shows two *L. harak* population clusters. The fish from Mozambique, South Africa, Madagascar, and Saudi Arabia formed a clade with a bootstrap value of 100%. The second clade comprised populations from Indonesia (Makassar and one other location), Japan, and Australia with a bootstrap value of 99%.

## Discussion

This confirms the view expressed by Baur and Leuenberger ^22^ that morphometric data can be extremely useful for the study of variation in form due to geographical differences. In addition, morphometric data are frequently used in taxonomy and fish description ^23^. In taxonomy, data on the holotype of a species are vital as a basis for comparison when observing other specimens. Data on the holotype can also be used to evaluate whether there are any changes in the morphometric characters of a given specimen. Such methods form the basis for establishing new fish species ^24^.

Their proximity indicates similarities in morphometric characters of *L. harak* between populations experiencing similar environmental conditions. The variation of the morphological characters of *L. harak* is largely determined by the level of destructive fishing and high fishing pressure ^23^. These changes can cause emperor fish spine abnormalities ^24^ Conversely, environmental pressures will tend to drive adaptation through physiological and behavioural modifications. A species with the capacity to adapt through plasticity will produce a variety of phenotypes as well as genotypes in response to a given set of environmental conditions, in order to increase fitness, the ability of individuals to survive and reproduce ^25, 26^. Matthews ^27^ found adaptations in body shape, colour and fins based on the environmental conditions in which fish live, as well as adaptations in head shape associated with feeding processes.

Genetic effects and environmental parameters can both result in differences in growth and development processes, which will produce variations in fish body shape ^28^. A relatively high level of geographical isolation coupled with limited geographical extent can result in significant differences in morphometric and genetic characters between stocks or populations of the same species due to a lack of gene-flow between populations ^29^. This is supported by the identification of cryptic lethrinid species with similar morphological characters by Healey ^30^, e.g. *L. nebulosus* and *L. mahsena* in the SWIO. This study found two clades, with the Seychelles separated from the other populations in the SWIO. The emergence of this phenomenon in the Seychelles could be related to high fishing pressure and habitat degradation. Wilson & Clarke ^31^ report that increasing exploitation and environmental pressures can cause a decline in fish stock abundance, as well as reductions in the average size, and detrimental genetic selection which can reduce potential fecundity and mean spawning size, alter sex ratios, and interspecific balances, as well as reducing genetic diversity.

The genetic distance can indicate the level of kinship between populations. Koh *et al.* ^32^ state that the lower the genetic distance between individuals, the closer their kinship and vice versa. According to Nei ^33^, genetic distance values can be categorised as low (0.010 - 0.099), moderate (0.1-0.99), or high (1.00-2.00). Such phylogenetic analyses can be used to construct detailed relationships between species and estimate the divergence between members of a family descended from a common ancestor ^34^.

This pattern indicates a divergence in genetic characteristics between *L. harak* populations in the Pacific and Indian Oceans. Williams *et al.* ^35^ found a genetic break between the Pacific and Indian Oceans in several invertebrate taxa. The topology of their phylogenetic reconstruction grouped northwestern Australia with the Indian Ocean for most taxa, although some taxa showed an affinity with Pacific Ocean populations, which they considered may be due to the oceanographic conditions around nine million years ago. Meanwhile, as in our study, eastern Australia populations were more closely related to Pacific Ocean populations.

The characteristics of the water masses and oceanographic currents in the two oceans influence the distribution and population connectivity of marine organisms, and may affect their physiology and exert differential selection pressures, thus potentially influencing genetic characteristics in several ways. Surface currents are a key factor in larval dispersal ^36^. As a protogynous hermaphrodite, *L. harak* undergoes a process of gonad differentiation associated with sex change from the female to terminal male phase ^6, 37^. As a fish with a pelagic spawning reproductive strategy, *L. harak* releases pelagic eggs which drift with other plankton ^36– 40^. The density of *L. harak* eggs is similar to or slightly less than that of sea water, so that they float at or close to the sea surface ^39^. After hatching the larvae remain planktonic for a period of time, which can vary from a few hours to several months ^40^. It is therefore likely that surface currents significantly affect the distribution and genetic population structure of *L. harak*.

The Pacific Low Latitude Western Boundary Currents (LLWBCs) are one of the factors that can influence genetic structure in the Indian Ocean ^41^. In the Pacific Ocean there are at least four currents affecting the distribution of marine organisms: the New Guinea Coastal Current (NGCC), the Halmahera Eddy (HE), the North Equatorial Coastal Current (NECC) and the North Equatorial Current (NEC) ^42^. The main source of the water masses underpinning current circulation in the LLWBCs system are the South Equatorial Current (SEC) and the North Equatorial Current (NEC) ^43^. A proportion of the water masses travelling westwards in the equatorial currents join the Kuroshio current flowing northward towards Japan, while some flows south in the Mindanao current or flows south to join the East Australia Current (EAC). A branch of the Mindanao current enters the Sulawesi Sea and flows through the Makassar Strait, while another branch flows east along the north coast of Sulawesi to the Halmahera Sea ^44^. These complex currents are further complicated by seasonal variations and local geography, and could enable gene-flow between several of the sites in our study.

Despite the clear demarcation of the two clades found in our study, the genetic distances between *L. harak* from the Pacific and Indian Oceans was relatively small, unlike the large differences found by Carvalho ^10^ between the Pacific and Atlantic populations of *Haemulon steindachneri*. These differences were considered sufficiently large to indicate that the two putative populations of *H. steindachneri* were in fact two different species, thus indicating speciation after the closing of the Isthmus of Panama. In contrast, despite the reduced connectivity between the Indian and Pacific Oceans described by Williams *et al.* ^35^, their data indicate some continuing gene-flow between the Pacific and Indian Oceans, although oceanographic flow patterns ^44^ indicate that gene-flow is more likely from the Pacific to the Indian Ocean than the reverse. Our results are consonant with such a continued (albeit reduced) gene-flow.

## Conclusion

The *Lethrinus harak* populations in the Pacific and Indian Oceans appear to form two major clades based on morphometric and genetic characteristics. One clade includes populations from South Africa, Mozambique, Madagascar and Saudi Arabia, while the other includes populations in Japan (Okinawa, Miyazaki, and Ishigaki), Indonesia (Makassar and Maluku) and the Philippines. Two populations exhibit intermediate (Australia) or unique (Seychelles) morphometric characters; however, based on the genetic reconstruction topology, the Australian population grouped with the Pacific *L. harak* clade.

## Data availability

### Underlying data

Partial cytochrome C oxidase subunit I (COX1) gene mitochondrial DNA sequence of *L. harak* (specimen voucher MSFUH0000591) from Makassar waters on GenBank, Accession number MT551656.
