# Supplementary material for: Morphological and genetic evaluation of the thumbprint emperor, Lethrinus harak (Forsskål, 1775) in the Pacific and Indian Oceans
Source: F1000Res. 2021 Mar 16;9:915. Originally published 2020 Aug 5. [Version 2] doi: 10.12688/f1000research.23740.2 (PMC8264806; doi:10.12688/f1000research.23740.2)
Supplement: Peer review history

# Peer review: Reviewer response for version 2

**DOI:** 10.5256/f1000research.54666.r88522

**Published:** 2021-07-16

**Authors:** Siti Azizah Mohd Nor

**Affiliations:** 1 Institute of Marine Biotechnology, Universiti Malaysia Terengganu, Terengganu, Malaysia

**Abstract**

 The main finding of highly structured populations of *L. harak* into the Pacific and Indian clusters should be emphasised clearly. The present conclusion is not representative of the major finding.

**Method**

 Specimens for morphometric analysis are not well defined. Most are described on Page 4 but others are under Figure 1. And apparently based on Table 1, those obtained from public databases (Page 5) for molecular analysis were also analysed for the morphometric analysis. This is rather puzzling? Did the databases provide photos (clarified ones) for measurements?  

- Just state the software used in this study (Page 5). There is no need to include those not relevant to this study.

DNA extraction protocol from museum-preserved sample is challenging and should be described .

 Results: Based on Figure 3, sample sizes are very low, not appropriate for DNA barcoding. At best it provides some insight into phylogeographic pattern based on COI gene which is a DNA barcoding gene, but not utilised in this context.

**Discussion**

 Authors need to relate their findings with the factors they describe. While these factors may be relevant, there is no attempt to incorporate the observations for example Pacific and Indian Ocean are isolated, attributed to a genetic barrier.

- The values for genetic distance should be converted to a percentage (%) which makes it 0 - 4 %. The maximum is indication of a different taxa (See Ward 2005 and other related articles). Thus, it is not correct to see all values are low (Page 9).

On the whole the data is a good preliminary contribution in this field of study, region and organism investigated and is publishable after addressing these comments.

Is the work clearly and accurately presented and does it cite the current literature?

Partly

If applicable, is the statistical analysis and its interpretation appropriate?

Yes

Are all the source data underlying the results available to ensure full reproducibility?

Partly

Is the study design appropriate and is the work technically sound?

Yes

Are the conclusions drawn adequately supported by the results?

Yes

Are sufficient details of methods and analysis provided to allow replication by others?

Partly

Reviewer Expertise:

Population  genetics and molecular ecology

I confirm that I have read this submission and believe that I have an appropriate level of expertise to confirm that it is of an acceptable scientific standard, however I have significant reservations, as outlined above.

## References

[1] : DNA barcoding Australia's fish species. *Philos Trans R Soc Lond B Biol Sci* .2005;360(1462) : 10.1098/rstb.2005.1716 1847-57 10.1098/rstb.2005.1716 16214743PMC1609232

# Peer review: Reviewer response for version 2

**DOI:** 10.5256/f1000research.54666.r88521

**Published:** 2021-07-07

**Authors:** Arvind Kumar Dwivedi

**Affiliations:** 1 Department of Habitat Ecology, Wildlife Institute of India, Dehradun, Uttarakhand, India

Reports on "Morphological and genetic evaluation of the thumbprint emperor, *Lethrinus harak* (Forsskål, 1775) in the Pacific and Indian Oceans"

 The study is important in understanding population structure of this important fish species at morphological and genetic level. However, I believe that authors should add more analytical aspects on morphological studies:

- Authors should include CVA or DFA, Thin plate Spline, Wireframes or Relative Warps in the method section for better interpretation of the results on morphological and shape analysis. DFA will provide more information about the discriminating variables between populations.
- After including analysis especially related to DFA and by including at least one visualization graph (Thin Plate or Wireframe), results of morphological study can be interpreted in a better way. 
- Morphological results need more support by incorporating suggested analysis to reach adequate conclusion.

After including the above suggestions, I believe the manuscript will be suitable for indexing.

Is the work clearly and accurately presented and does it cite the current literature?

Yes

If applicable, is the statistical analysis and its interpretation appropriate?

No

Are all the source data underlying the results available to ensure full reproducibility?

No source data required

Is the study design appropriate and is the work technically sound?

Yes

Are the conclusions drawn adequately supported by the results?

Partly

Are sufficient details of methods and analysis provided to allow replication by others?

No

Reviewer Expertise:

Fish Morphometrics at various levels (Traditional, Truss and Geometric Morphometrics)

I confirm that I have read this submission and believe that I have an appropriate level of expertise to confirm that it is of an acceptable scientific standard.

# Peer review: Reviewer response for version 2

**DOI:** 10.5256/f1000research.54666.r81546

**Published:** 2021-05-21

**Authors:** Femy Mahmud Sahami

**Affiliations:** 1 Department of Aquatic Resources Management, Gorontalo State University, Gorontalo, Indonesia

This research article with tittle "Morphological and genetic evaluation of the thumbprint emperor, *Lethrinus harak* (Forsskål, 1775) in the Pacific and Indian Oceans" has been reviewed.  For this Version 2, there is no major or minor revision needed in this article. This is appropriate, and I approve this research article.

Is the work clearly and accurately presented and does it cite the current literature?

Partly

If applicable, is the statistical analysis and its interpretation appropriate?

Partly

Are all the source data underlying the results available to ensure full reproducibility?

Partly

Is the study design appropriate and is the work technically sound?

Partly

Are the conclusions drawn adequately supported by the results?

Partly

Are sufficient details of methods and analysis provided to allow replication by others?

Yes

Reviewer Expertise:

Goby fish

I confirm that I have read this submission and believe that I have an appropriate level of expertise to confirm that it is of an acceptable scientific standard.

# Peer review: Reviewer response for version 1

**DOI:** 10.5256/f1000research.26194.r74360

**Published:** 2020-11-19

**Authors:** Femy Mahmud Sahami

**Affiliations:** 1 Department of Aquatic Resources Management, Gorontalo State University, Gorontalo, Indonesia

- The conclusion in abstracts is still too general. The research novelty needs to be explicitly highlighted, particularly the differences of *L. harak* in the Pacific and Indian oceans based on morphometric and genetic aspects.
- The word “alcohol” was mentioned twice in the following sentences: Each sample was placed on its right-hand side and sterilised using alcohol.  A fin-clipping of around 1.8 cm in length was taken from the pectoral fin and inserted into a 2 ml micro tube 2 filled with 90% alcohol. Please consider replacing “alcohol” with “ethanol” to avoid misinterpretation.
- The method described as: A fin-clipping of around 1.8 cm in length was taken from the pectoral fin and inserted into a 2 ml micro tube 2 filled with 90% alcohol. This sentence needs to elaborate in detail. Please connect it with the different-samples explanation in the DNA extraction section.
- It is not clear how many samples were taken in Makassar for morphometric and molecular analysis purposes. Samples with the code MSFUH000591, MSFUH0000592, MSFUH0000593 were not specified what analysis was applied. Only the sample coded MSFUH000591 was mentioned in the results section. Likewise, in Table 1, there were stated only 3 Makassar samples with the code MSFUH000591, MSFUH915, and MSFHU917. Where were the other samples (coded MSFUH0000592 and MSFUH0000593) descriptions?
- In the method mentioned: One specimen had been collected from Meitsu, Nago, Miyazaki, Japan (MUFS 16284) and one from the Philippines (MUFS 6136) (Figure 1). However, these two samples were not shown in Figure 1. What is the purpose of writing “(Figure 1)” at the end of the sentence?
- What do “preceding samples” mean in the following sentence “The preceding samples were used in this study to represent the Pacific Ocean”
- Two sentences seem somewhat ambiguous in measuring morphology, as follows: Measurements of these remotely sourced specimens were made using ImageJ version 1.52a software14, except for the interorbital width character, which could not be measured from the downloaded photographs. Then in the following sentence, it was stated that: The characters measured using callipers (accuracy 0.02 mm) included standard length, body depth, head length, pectoral length, pelvic length, orbital length, interorbital width, snout length, suborbital width, upper jaw length. It might be necessary to clarify which samples were measured using ImageJ and measured manually using a caliper.
- The second sentence's meaning in the DNA extraction sub-section is not clear: Fin clippings (2–3cm) were taken from the pectoral fin of each specimen using surgical scissors or a surgical scalpel. Each sample was preserved in a labelled 2ml tube filled with 95% ethanol. Are the specimen referred to samples from Makassar or some other areas? As in the 6th sentence in the Fish Specimen sub-section stated as: A fin-clipping of around 1.8 cm in length was taken from the pectoral fin and inserted into a 2 ml micro tube 2 filled with 90% alcohol.
- If possible, replace the subsection “Statistical Analysis” with “Data Analysis” since there were also explaining genetic analysis.
- In the second paragraph in the sub-section Morphological Characters, it mentioned “Indonesia (Makassar and Maluku)”, while in Figure 1 it was stated that: Specimens of Lethrinus harak. A. Specimen JN311937, paratype, SL 170mm, collected from Arumbai Fish Market (-3°68’N, 128°18’E), Ambon, Indonesia, 06 January 2016. What mentioned here was Ambon, not Maluku. The words Maluku and Ambon need to be consistent as even though Ambon is in Maluku, this will lead to confusion. Also, Figure 2 mentioned only Makassar and Indonesia, while Maluku was not mentioned. In Table 1 and Table 2, the word Maluku was not mentioned either, but in conclusion, it was written in Indonesia (Makassar and Maluku). Please make it consistent whether Indonesia (Maluku) or Indonesia (Ambon).
- It needs consistency in writing. It should be written in capital letters at the beginning of each word in the stands: The Pacific low latitude western boundary currents (LLWBCs).
- The writing of citations is still inconsistent. Some are written with the author's name along with the serial number in the bibliography, and some are written directly without writing the author's name.

Is the work clearly and accurately presented and does it cite the current literature?

Partly

If applicable, is the statistical analysis and its interpretation appropriate?

Partly

Are all the source data underlying the results available to ensure full reproducibility?

Partly

Is the study design appropriate and is the work technically sound?

Partly

Are the conclusions drawn adequately supported by the results?

Partly

Are sufficient details of methods and analysis provided to allow replication by others?

Yes

Reviewer Expertise:

Goby fish

I confirm that I have read this submission and believe that I have an appropriate level of expertise to confirm that it is of an acceptable scientific standard, however I have significant reservations, as outlined above.

# Response

**Published:** 2020-11-27

**Authors:** Muhammad Afrisal

**Affiliations:** Universitas Hasanuddin, Indonesia

Thank you for being our manuscript reviewer. We will revise your suggestions for writing our manuscript.

# Peer review: Reviewer response for version 1

**DOI:** 10.5256/f1000research.26194.r68802

**Published:** 2020-08-26

**Authors:** Jayasankar Pallipuram

**Affiliations:** 1 Marine Biotechnology Division, ICAR-Central Marine Fisheries Research Institute, Kochi, Kerala, India

No justification is being given for using alternative software used for genetic analysis – without guaranteeing total equivalence, two different softwares are used [page 5]

 Though sample details are furnished, it hasn’t been clearly mentioned anywhere as to exact number of samples subjected to DNA barcoding. However, from the report the number seems to be insufficient to interpret the results.

 If DNA were extracted from museum-preserved samples, the methodology used should have been specifically mentioned. The protocol is certainly different from that used for fresh/frozen samples.

 In the methodology it’s mentioned “Amplified PCR products with a length (in bp) consonant with the target gene segment were sent to a sequencing company (1st Base Asia, Singapore) in order to obtain DNA sequence data.” Though we accept that in majority of studies DNA sequencing is outsourced, such kind of statement as given here would dilute the scientific seriousness and originality. The authors haven’t even briefly described the actual methodology of DNA sequencing as intended and executed. 

 In discussion the authors have described endlessly on influence of water currents and other environmental parameters in stock distribution and separation. However, the present study has no such data to substantiate those concepts. This has resulted in just copying others’ observations and presenting the same, without having any study attempted in those lines by the authors.

Is the work clearly and accurately presented and does it cite the current literature?

Partly

If applicable, is the statistical analysis and its interpretation appropriate?

Partly

Are all the source data underlying the results available to ensure full reproducibility?

Partly

Is the study design appropriate and is the work technically sound?

Partly

Are the conclusions drawn adequately supported by the results?

Partly

Are sufficient details of methods and analysis provided to allow replication by others?

No

Reviewer Expertise:

Aquaculture, fish genetics, molecular taxonomy

I confirm that I have read this submission and believe that I have an appropriate level of expertise to confirm that it is of an acceptable scientific standard, however I have significant reservations, as outlined above.
